# Lithium ion trapping mechanism of SiO_2_ in LiCoO_2_ based memristors

**DOI:** 10.1038/s41598-019-41508-3

**Published:** 2019-03-25

**Authors:** Qi Hu, Runmiao Li, Xinjiang Zhang, Qin Gao, Mei Wang, Hongliang Shi, Zhisong Xiao, Paul K. Chu, Anping Huang

**Affiliations:** 1 School of Physics, Beihang University, Beijing, 100191 China; 2 Department of Physics and Department of Materials Science and Engineering, City University of Hong Kong, Tat Chee Avenue, Kowloon, Hong Kong China

## Abstract

Pt/LiCoO_2_/SiO_2_/Si stacks with different SiO_2_ thicknesses are fabricated and the influence of SiO_2_ on memristive behavior is investigated. It is demonstrated that SiO_2_ can serve as Li ion trapping layer benefiting device retention, and the thickness of SiO_2_ must be controlled to avoid large SET voltage and state instability. Simulation model based on Nernst potential and diffusion potential is postulated for electromotive force in LiCoO_2_ based memristors. The simulation results show that SiO_2_ trapping layer decreases the total electromotive field of device and thereby prevents Li ions from migrating back to LiCoO_2_. This model shows a good agreement with experimental data and reveals the Li ion trapping mechanism of SiO_2_ in LiCoO_2_ based memristors.

## Introduction

Boasting high memory density and energy efficiency, memristors are promising alternatives to complementary metal oxide semiconductors (CMOS) for high-density storage and high-performance computing^1–3^. Neural networks based on crossbar arrays of filamentary memristors or phase change memories have been reported and successfully implemented in image recognition and word classification^4–9^. Despite significant progresses, these devices still suffer from several limitations such as excessive “write” noise as well as high switching voltages and currents^10–12^. LiCoO_2_ based memristors are expected to overcome several limitations. A major advantage of LiCoO_2_ based memristors is that the intercalation and extraction of Li ions in LiCoO_2_ are highly reversible leading to high device stability^13,14^. Moreover, Li ions migration in LiCoO_2_ based memristors is similar to information exchange processes between synapses and neurons in the brain^15^. With a low energy barrier for Li ions transporting, LiCoO_2_ based memristors have a smaller threshold voltage and are expected to satisfy low-power consumption requirement in high performance computing^16,17^.

Generally, LiCoO_2_ based memristors operate by a common electrochemical reaction between LiCoO_2_ and Si^18,19^. The conductivity of LiCoO_2_ changes as a function of Li ions concentration^20,21^. Under a positive electrical field, Li ions migrate from LiCoO_2_ to Si and the variation of Li ions concentration in LiCoO_2_ produces a resistive switching (RS) behavior. The RS processes corresponding to Li ions migration out of Li-based oxide have been experimentally verified^22,23^. Furthermore, the electrochemical reaction between LiCoO_2_ and Si produces an electromotive force (EMF) which in LiCoO_2_ based memristors can cause electrical short circuit between Li_x_CoO_2_ and Li_x_Si decreasing device retention^24^. It has been reported that SiO_2_ between LiCoO_2_ and Si can work as solid state electrolyte allowing transport of Li ions and trap Li ions when external voltage is removed thus increasing device retention^23,24^. SiO_2_ has been also reported to be a promising candidate for the electrolyte or controllable barrier layer in CMOS and Li-ion batteries, which can be used to modulate the Li ions transporting^23–27^. However, the influence of SiO_2_ on memristive behavior in LiCoO_2_ based memristors has not been investigated and the Li ion trapping mechanism of SiO_2_ in LiCoO_2_ based memristors remains to be revealed.

In this work, Pt/LiCoO_2_/SiO_2_/Si stacks with different SiO_2_ thicknesses are produced and the corresponding memristive properties such as electrical properties, stabilities and retentions are investigated. SiO_2_ serves as a trapping layer for Li ions and is favorable for device retention. It is also necessary to control the SiO_2_ thickness to an appropriate range for higher durability and state stability. A simulation model for EMF in LiCoO_2_ based memristors is proposed to explain the influence of SiO_2_ on Pt/LiCoO_2_/SiO_2_/Si stacks. The origins of EMF include Nernst potential and diffusion potential. Thus, this model is based on Nernst potential and diffusion potential and the total electromotive fields of device are calculated. It can be seen that SiO_2_ trapping layer can decrease electromotive field of Pt/LiCoO_2_/SiO_2_/Si stacks. This model is consistent with the experimental results and reveals the Li ion trapping mechanism of SiO_2_ in LiCoO_2_ based memristors.

## Methods

The 40 nm LiCoO_2_ films were deposited on highly doped p-type Si (111) or SiO_2_/Si substrates by pulse laser disposition (PLD) using a stoichiometric LiCoO_2_ target. LiCoO_2_ layer was fabricated in O_2_ (10 Pa) atmosphere at 550 °C to obtain R-3m LiCoO_2_ phase through a KrF laser (LightMachinery IPEX-800, λ = 248 nm and τ = 25 ns) operated at 3 Hz with a fluence of ≈1.3 J cm^−2^. SiO_2_ layers were formed by thermal oxidization in an oxygen environment at 900 °C. The thickness of SiO_2_ layer was determined by profilometry. The SiO_2_ layers had thicknesses of 10 nm, 20 nm and 40 nm (${d}_{Si{O}_{2}}:\,{d}_{LiCo{O}_{2}}=1:\,4,\,1:\,2,\,1:\,1$) in order to investigate the influence of the SiO_2_ thickness on the memristive behavior of Pt/LiCoO_2_/SiO_2_/Si stacks. The LiCoO_2_ deposition was carried out under same conditions to obtain same Li ions concentration and layer thickness. To avoid introducing other metal ion such as Ag^+^ or Cu^2+^, noble Pt was used as the top electrodes. The 80 nm thick Pt top electrodes were prepared by magnetron sputtering in pure Ar with a metal mask covering the LiCoO_2_ layer. The diameter of the top electrode was 1 mm.

The electrical and memristive properties were measured by the voltage sweeping mode on Keithley 4200-SCS semiconductor parameter analyzer at room temperature. The voltages were applied to the Pt top electrode with Si substrate grounded. The cycle tests were collected continually by the direct-current (DC) voltage sweeping mode. The retention tests were conducted after the devices switching to LRS, and the read voltage is 0.1 V. The LiCoO_2_ layers were analyzed with a LabX XRD-6000 using Cu Kα radiation and operating at 40 kV and 30 mA. The scanning rate was 5°/min.

## Results

**Figure 1:**
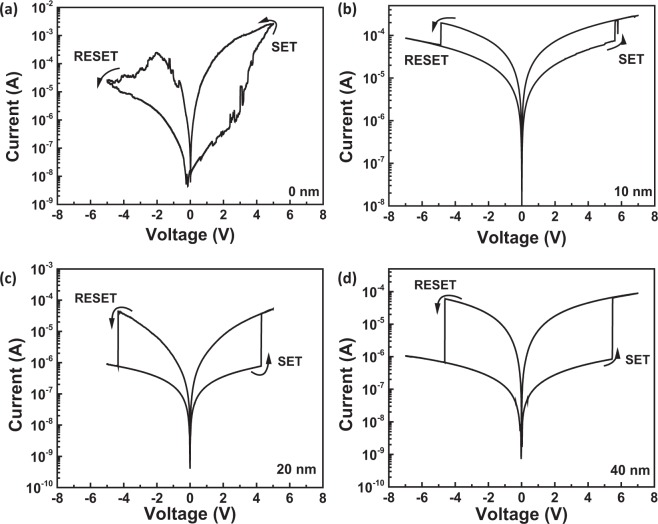
I-V curves of samples with different SiO_2_ thicknesses.

The Pt/LiCoO_2_/SiO_2_/Si stacks with different SiO_2_ thicknesses of 10 nm, 20 nm and 40 nm are fabricated and samples without SiO_2_ are also prepared for comparison. The corresponding I-V curves of different samples are shown in Fig. 1. The I-V curve of sample (a) displays gradual RS processes without definite V_set_ and V_reset_, corresponding to homogeneous RS in LiCoO_2_^24^. On the contrary, samples (b), (c) and (d) exhibit abrupt current increases similar to RS behavior of electrochemical metallization (ECM)^10^, as shown in Fig. 1(b–d) (More I-V curves during cycle tests are shown in supporting information, Fig. S3). Hence, samples with and without SiO_2_ characterize different memristive properties which may be attributed to different transport processes of Li ions in SiO_2_ and LiCoO_2_.

**Figure 2:**
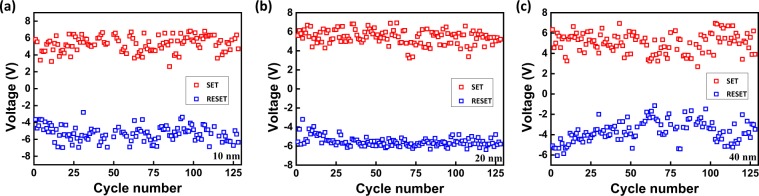
V_SET_ and V_RESET_ of samples with different SiO_2_ thicknesses (128 cycles).

**Figure 3:**
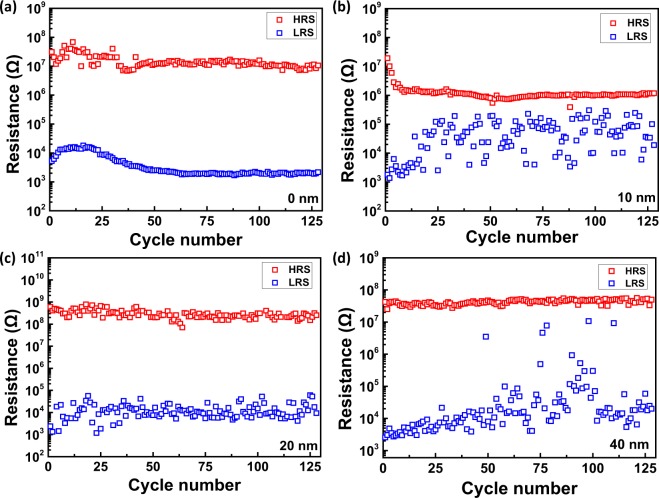
R_HRS_ and R_LRS_ of samples with different SiO_2_ thicknesses (128 cycles).

In order to investigate the device stabilities, durability tests are conducted on the different samples. As shown in Fig. 2, the V_SET_ and V_RESET_ of samples with 20 nm are more stable. To evaluate the dispersion degree, corresponding coefficients of variation (C_V_) are calculated. C_V_ is defined by^28^

1 $${{\rm{C}}}_{V}=\frac{\sigma }{\mu }$$

where σ is the standard deviation and μ is the average of several data. The ranges of SET voltage and C_V_ of different samples are summarized in Table 1 (For each SiO_2_ thickness, 15 points of the sample are measured). Samples with 20 nm show smaller C_V_ than other samples consistent with Fig. 2. To study state stability, the resistances of different samples at HRS and LRS for 128 cycles are also measured. Figure 3 plots the R_HRS_ and R_LRS_ of different samples during 128 cycles. The R_HRS_ of samples (a) and (b) show two orders of magnitude fluctuations while those of samples (c) and (d) are within one order of magnitude. The R_LRS_ of sample (c) shows the smallest fluctuation. The C_V_ of R_HRS_ and R_LRS_ are also calculated in Table 2 (For each SiO_2_ thickness, 15 points of the sample are measured). As the SiO_2_ thickness is increased, C_V_ of R_HRS_ decreases and sample (c) has the smallest C_V_ of R_LRS_. However, sample (d) shows several RS failures during the durability test. Hence, samples with 20 nm SiO_2_ characterize the best stability. SiO_2_ with appropriate thickness is favorable for state stability but SiO_2_ that is too thick decreases the state stability.

**Table 1:** The SET voltage, RESET voltage and corresponding coefficients of variation (C_V_) of in samples with different SiO_2_ thickness.

Thickness (nm)	V_SET_ (V)	V_SET_ C_V_	V_RESET_ (V)	V_RESET_ C_V_
0	—	—	—	—
10	2.62~6.82	0.17	−2.81~−6.97	−0.07
20	3.24~6.93	0.14	−3.2~−6.38	−0.08
40	3.42~6.21	0.18	−2.03~−4.28	−0.23

**Table 2:** The HRS, LRS and corresponding coefficients of variation (C_V_) of in samples with different SiO_2_ thicknesses.

Thickness (nm)	HRS (Ω)	HRS C_V_	LRS (Ω)	LRS C_V_
0	10^7^~10^8^	0.59	10^3^~10^4^	0.97
10	10^6^~10^7^	0.46	10^3^~10^5^	1.01
20	10^8^	0.44	10^3^~10^4^	0.80
40	10^7^	0.13	10^3^~10^6^	0.95

**Figure 4:**
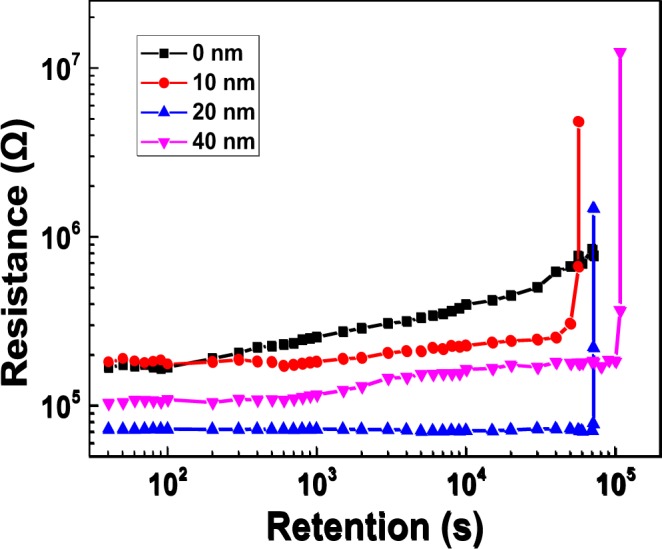
Retention tests of samples with different SiO_2_ thicknesses at a read voltage of 0.1 V.

Retention of Pt/LiCoO_2_/SiO_2_/Si stacks with different SiO_2_ thicknesses is also assessed at a read voltage of 0.1 V to evaluate ability of data storage. Figure 4 shows that Pt/LiCoO_2_/SiO_2_/Si stacks without SiO_2_ maintain retention characteristic of ~10^3^ s whereas samples with SiO_2_ show good retention characteristics up to 10^5^ s. As SiO_2_ thickness is increased, device retention increases from 10^4^ to 10^5^ s. In terms of Pt/LiCoO_2_/SiO_2_/Si stacks, EMF mainly drives Li ions from Si to LiCoO_2_ without an external voltage and therefore, SiO_2_ can decrease the influence of EMF on Pt/LiCoO_2_/SiO_2_/Si stacks leading to high retention.

## Discussion

**Figure 5:**
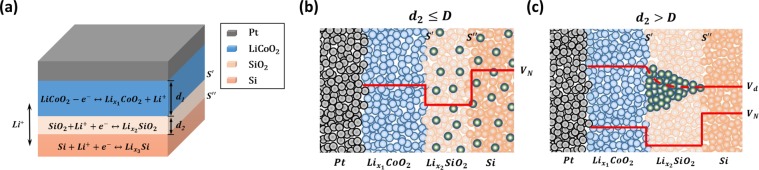
(**a**) Schematic of Pt/LiCoO_2_/SiO_2_/Si stacks and Li ions transportation processes; (**b**) Origins of electromotive force (EMF) in Pt/LiCoO_2_/SiO_2_/Si stacks (d_2_ **≤** D); (**c**) Origins of EMF in Pt/LiCoO_2_/SiO_2_/Si stacks (D < d_2_)

To understand the mechanism of trapping of Li ions in SiO_2_, it is necessary to investigate the Li ions transporting processes in the Pt/LiCoO_2_/SiO_2_/Si stacks. The schematic of Pt/LiCoO_2_/SiO_2_/Si stacks and Li ions transporting processes are shown in Fig. 5(a). The Li ions transporting processes in LiCoO_2_, SiO_2_ and Si have been experimentally verified and reported for Li-ion battery^22,24–26^. The electrochemical reactions involving Li ions in Pt/LiCoO_2_/SiO_2_/Si stacks are as follows:

2 $$LiCo{O}_{2}-(1-{x}_{1}){e}^{-}\leftrightarrow L{i}_{{x}_{1}}Co{O}_{2}+(1-{x}_{1})L{i}^{+}$$

3 $$Si{O}_{2}+{x}_{2}L{i}^{+}+{x}_{2}{e}^{-}\leftrightarrow L{i}_{{x}_{2}}Si{O}_{2}$$

4 $$Si+{x}_{3}L{i}^{+}+{x}_{3}{e}^{-}\leftrightarrow L{i}_{{x}_{3}}Si$$

At a positive voltage, Li ions are extracted from LiCoO_2_ and migrate in the interval positions of SiO_2_ finally forming Li_x_Si in Si substrate. The LiCoO_2_ acts as Li ions source and RS layer, SiO_2_ allows Li ions transportation, and Si is used to store Li ions. The LiCoO_2_ layers are annealed at 550 °C to obtain R-3m phase (seen in Fig. S1) which is hexagonal layered structure with a uniform Li ions distribution^19,20^. Owing to the uniform Li ions distribution in crystalline R-3m LiCoO_2_, the RS processes occur in the entire LiCoO_2_ layer displaying gradual current changes. In contrast to layered structure LiCoO_2_, SiO_2_ is amorphous with non-uniformity, and the Li ions intercalation processes are inhomogeneous and occur in some partial regions of SiO_2_. Therefore, the memristive behavior of samples without SiO_2_ shows gradual current changes while samples with SiO_2_ exhibit abrupt resistance jumps. Furthermore, it has been reported in Li-ion batteries that insertion of Li ions in Si strongly strains the crystalline lattice thus harming the device stability, the SiO_2_ can work as a buffer layer to decrease the crystalline lattice stress^22,29^ and trap Li ions decreasing resistance degradation without an external voltage^23,24^. However, thin SiO_2_ layer (≤10 nm) is generally rough exhibiting non-uniformity^27^, which may be adverse to device stability as shown in Fig. 3(b). And too thick SiO_2_, such as 40 nm, exhibit stronger Li ion trap effect which can cause several set failures and reduced performances, as shown in Fig. 3(d). Hence, SiO_2_ with the appropriate thickness improves the device stability.

**Figure 6:**
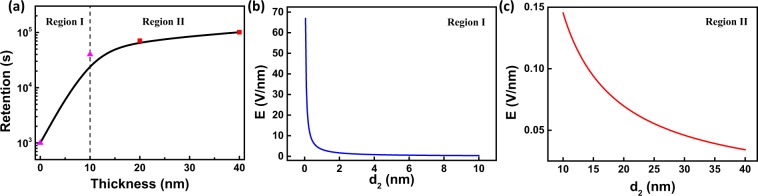
(**a**) Experimental device retention as a function of SiO_2_ thickness (2 different regions are observed); (**b**) Simulated E-d_2_ characteristics in Pt/LiCoO_2_/SiO_2_/Si stacks without an external voltage (d_2_ ≤ 10 nm); (**c**) Simulated E-d_2_ characteristic in Pt/LiCoO_2_/SiO_2_/Si stacks without an external voltage (10 nm < d_2_ ≤ 40 nm)

After applying a positive voltage, Li ions transport from LiCoO_2_ to Si resulting in EMF between LiCoO_2_/SiO_2_ interface ($s^{\prime}$) and SiO_2_/Si interface ($s^{\prime\prime}$). Two factors mainly contribute to the EMF: (a) Nernst potential V_N_ and (b) diffusion potential V_d_^30^. SiO_2_ works as a Li ion trapping layer allowing Li ions to transport at a positive voltage while trapping Li ions and avoiding resistance degradation without an external voltage. According to the different SiO_2_ thickness (d_2_), two origins of EMF in Pt/LiCoO_2_/SiO_2_/Si stacks are shown in Fig. 5(b) and (c). When *d*_2_ ≤ *Critical poin (D)*, SiO_2_ undergoes fully lithiation and exhibits homogeneous Li ions distribution, as shown in Fig. 5(b), and V_EMF_ = V_N_. When *d*_2_ > *D*, the SiO_2_ acts as a RS layer and has an inhomogeneous Li ions distribution as shown in Fig. 5(c), V_EMF_ = V_N_ + V_d_. Therefore, the influence of SiO_2_ trapping layer on EMF can be divided into two regions. According to the range of x_1_ in ${{\rm{Li}}}_{{{\rm{x}}}_{1}}{{\rm{CoO}}}_{2}$ and x_2_ in ${{\rm{Li}}}_{{{\rm{x}}}_{2}}{{\rm{SiO}}}_{2}$, the range of D between region I and region II can be calculated to be $6.96\,{\rm{nm}} < {\rm{D}} < 13.91\,\mathrm{nm}\,$(Calculation processes are shown in Supplementary Information). Figure 6(a) shows different tendencies in 0~10 nm region and 10~40 nm region. When $0\,\mathrm{nm} < {{\rm{d}}}_{2}\le 10\,\mathrm{nm}$, the device retention rises rapidly with increasing SiO_2_ thickness and when $10\,\mathrm{nm} < {{\rm{d}}}_{2}\le 40\,\mathrm{nm}$, device retention rises slightly with increasing SiO_2_ thickness, indicating that D is near to 10 nm which is consistent with the calculation. This can also explain the abnormal tendency of HRS in Figs. 1 and 3. The HRS of devices mainly consist of ${{\rm{R}}}_{{{\rm{Li}}}_{{\rm{x}}}{{\rm{CoO}}}_{2}}$ and ${{\rm{R}}}_{{{\rm{Li}}}_{{\rm{y}}}{{\rm{SiO}}}_{2}}$. For samples without SiO_2_, Li ions transport back to LiCoO_2_ after reset process and ${{\rm{R}}}_{{\rm{HRS}}}\approx {{\rm{R}}}_{{{\rm{LiCoO}}}_{2}}$. For samples with SiO_2_, the SiO_2_ would trap large amount of Li ions during reset process resulting in decrease of Li ion concentration in LiCoO_2_ and the retained Li ions in SiO_2_ enhance the conductivity. Therefore, the HRS of Pt/LiCoO_2_/SiO_2_ (10 nm)/Si is lower than Pt/LiCoO_2_/Si. Moreover, 10 nm SiO_2_ undergoes full lithiation while 20 nm and 40 nm SiO_2_ undergo partly lithiation. Full lithiated SiO_2_ would retain higher Li ion concentration and cause lower Li ion concentration in LiCoO_2_ and higher conductivity. Furthermore, thicker SiO_2_ means larger Li ion storage and stronger Li ion trap effect leading to lower Li ion concentration in LiCoO_2_, which may cause that the HRS of samples with 40 nm SiO_2_ is lower than that of samples with 20 nm SiO_2_.

The device retention is mainly relative to the electromotive field (E = V_EMF_/d_2_), thus the E-d_2_ characteristics are discussed. In region I, SiO_2_ behaves as solid-state electrolyte exhibiting a homogeneous Li ions distribution and V_EMF_ = V_N_. EMF drives Li ions migration from Li_x_Si to Li_x_CoO_2_ and the main electrochemical reactions are as follows:

5 $$C{o}^{4+}+{e}^{-}\leftrightarrow C{o}^{3+}(Interface\,s^{\prime} )$$

6 $$L{i}^{+}+{e}^{-}\leftrightarrow Li(Interface\,s^{\prime\prime} )$$

The Nernst potential is given by^30^:

7 $${V}_{N}={V}_{s^{\prime} }-{V}_{s^{\prime\prime} }={V}^{0}+\frac{kT}{e}ln\frac{{a}_{C{o}^{4+}}\cdot {a}_{Li}}{{a}_{C{o}^{3+}}\cdot {a}_{L{i}^{+}}}$$

${V}^{0}$ is the difference in the standard potentials of these reactions, *k* is Boltzmann constant, *T* is temperature, *e* is electron charge, ${a}_{{M}^{z+}}$ denote the activity of the cations (*a* = *γc*, *γ* is activity coefficient). The amount of Li ions in Pt/LiCoO_2_/SiO_2_/Si stacks is constant:

8 $${c}_{0}{d}_{1}S={c}_{1}{d}_{1}S+{c}_{2}{d}_{2}S+{c}_{3}{d}_{3}S$$

where *c*_0_ denotes the initial concentration of Li ions in LiCoO_2_, *c*_1_, *c*_2_ and *c*_3_ are the concentration of Li ions in Li_x_CoO_2_, SiO_2_ and Si, *S* is the cell size. *d*_1_, *d*_2_ and *d*_3_ are the thicknesses of LiCoO_2_, SiO_2_ and Si, respectively. The concentration of Li ions can be defined as $\,c=\frac{\frac{\rho V}{M}}{V}=\frac{\rho }{M}$ ($\rho$ is mass density, V is volume and M is the molar mass). Equation (8) can be written by:

9 $$\frac{{\rho }_{1}}{{M}_{1}}{d}_{1}=\frac{{\rho }_{1}}{{M}_{1}}{d}_{1}{x}_{1}+\frac{{\rho }_{2}}{{M}_{2}}{d}_{2}{x}_{2}+\frac{{\rho }_{3}}{{M}_{3}}{x}_{3}{d}_{3}$$

where *ρ*_1_, *ρ*_2_ *and ρ*_3_ are mass densities of LiCoO_2_, SiO_2_ and Si, respective. *M*_1_, *M*_2_ *and M*_3_ are molar mass of LiCoO_2_, SiO_2_ and Si, respectively. *x*_1_, *x*_2_ and *x*_3_ are the atomic percent in $L{i}_{{x}_{1}}Co{O}_{2}$, $L{i}_{{x}_{2}}Si{O}_{2}$ and $L{i}_{{x}_{3}}Si$. Due to low concentration of Li ions in LiCoO_2_ and SiO_2_ and small molar mass of lithium, the influences of Li ions on $\rho \,and\,M$ are neglected. Thus, ${c}_{C{o}^{4+}}=\frac{{\rho }_{1}}{{M}_{1}}(1-{x}_{1})$, ${c}_{C{o}^{3+}}=\frac{{\rho }_{1}}{{M}_{1}}{x}_{1}$, ${c}_{L{i}^{+}}=\frac{{\rho }_{2}}{{M}_{2}}{x}_{2}$, ${c}_{Li}=\frac{{\rho }_{3}}{{M}_{3}}{x}_{3}$. Based on Equation (9) and γ assumed to be one, the electromotive field in region I (E_1_ = V_EMF_/d_2_) can be calculated by:

10 $${E}_{1}=\frac{{V}^{0}}{{d}_{2}}+\frac{kT}{e{d}_{2}}ln\frac{(1-{x}_{1})[\frac{{\rho }_{1}}{{M}_{1}}{d}_{1}(1-{x}_{1})\,-\,\frac{{\rho }_{2}}{{M}_{2}}{d}_{2}{x}_{2}]}{{x}_{1}{d}_{3}\cdot \frac{{\rho }_{2}}{{M}_{2}}{x}_{2}}$$

To facilitate the discussion of EMF, reference values are chosen according to previous reports, ${x}_{1}=0.7$, ${\rho }_{1}=2.5\,g/c{m}^{3}$, ${\rho }_{2}=2.648\,g/c{m}^{3}$, ${\rho }_{3}=2.329\,g/c{m}^{3}$, ${M}_{1}=97.87\,g/mol$, ${M}_{2}=60.086\,g/mol$,$\,{M}_{3}=28.085\,g/mol$, ${V}^{0}=3.6\,V$, ${x}_{2}=2/3$^13,21,31,32^. The thickness of different layers are measured, ${d}_{1}=40\,nm$, ${d}_{3}=550\,{\rm{\mu }}m$. D is simulated to be 10.43 nm, and the simulated E-d_2_ characteristics are shown in Fig. 6(b). E_1_ decreases rapidly with d_2_, and thereby device retention rises rapidly with increasing SiO_2_ thickness.

In region II, SiO_2_ acts as a RS layer and V_EMF_ = V_N_ + V_d_. The main electrochemical reactions are as follows:

11 $$C{o}^{4+}+{e}^{-}\leftrightarrow C{o}^{3+}(Interface\,s^{\prime} )$$

12 $$S{i}^{4+}+{e}^{-}\to S{i}^{3+}\,(Interface\,{s}^{^{\prime\prime} })$$

Nernst potential is given by^30^

13 $${V}_{N}={V}_{s^{\prime} }-{V}_{s^{\prime\prime} }={V}^{0}+\frac{kT}{e}ln\frac{{a}_{C{o}^{4+}}\cdot {a}_{S{i}^{3+}}}{{a}_{C{o}^{3+}}\cdot {a}_{S{i}^{4+}}}$$

Similarly, the amount of Li ions in Pt/LiCoO_2_/SiO_2_/Si stacks is constant:

14 $${c}_{0}{d}_{1}S={c}_{1}{d}_{1}S+{c}_{2}{d}_{2}S$$

It can be also written as:

15 $$\frac{{\rho }_{1}}{{M}_{1}}{d}_{1}(1-{x}_{1})=\frac{{\rho }_{2}}{{M}_{2}}{d}_{2}{x}_{2}$$

The Li ions distribution in SiO_2_ is inhomogeneous, to simply the Li ions distribution in SiO_2_, it is assumed that Li ions in SiO_2_ follows constant-total-dopant diffusion and the Li ions distribution in SiO_2_ can be defined by^33^:

16 $$c(y,t)=\frac{Q}{\sqrt{\pi D^{\prime} t}}\exp (\,-\,\frac{{y}^{2}}{4D^{\prime} t})$$

where Q is dopant number of average area, *D*′ is the diffusion coefficient, t is the diffusion time and y is the depth. Assuming diffusion length is equal to SiO_2_ thickness $(L=\sqrt{{D}^{\text{'}}{t}_{1}}={d}_{2})$, the total dopant is the amount of Li ions extracted from LiCoO_2_, thus $Q=(1-{x}_{1})\,\frac{{\rho }_{1}}{{M}_{1}}{d}_{1}$. Herein, ${c}_{C{o}^{3+}}=\frac{{\rho }_{1}}{{M}_{1}}{x}_{1}$, ${c}_{C{o}^{4+}}=\frac{{\rho }_{1}}{{M}_{1}}(1-{x}_{1})$, ${c}_{S{i}^{3+}}={c}_{L{i}^{+}}\,=$ $c({d}_{2},{t}_{1})=\frac{(1-{x}_{1})\,\frac{{\rho }_{1}}{{M}_{1}}{d}_{1}}{\sqrt{\pi }{d}_{2}}\exp (\,-\,\frac{1}{4})$, ${c}_{S{i}^{4+}}=\frac{{\rho }_{2}}{{M}_{2}}-{c}_{S{i}^{3+}}$. Based on Equations (15, 16) and assuming γ = 1, the electromotive field (E_N_) originating from Nernst potential can be calculated by:

17 $${E}_{N}=\frac{{V}^{0}}{{d}_{2}}+\frac{kT}{e{d}_{2}}ln\frac{{\rho }_{1}{M}_{2}{d}_{1}{(1-{x}_{1})}^{2}\exp (\,-\,\frac{1}{4})}{\,{x}_{1}[\sqrt{\pi }{\rho }_{2}{M}_{1}{d}_{2}-\exp (\,-\,\frac{1}{4}){\rho }_{1}{M}_{2}{d}_{1}(1-{x}_{1})]}$$

In region II, V_d_ should be also taken in account. The diffusion potential formula is given by^30^:

18 $${V}_{d}=-\,\frac{kT}{e}\bar{t}ln\frac{{a}_{{s}^{\text{'}}}}{{a}_{{s}^{\text{'}\text{'}}}}$$

where $\bar{t}$ is ion transference number averaged throughout layer thickness, ${a}_{{s}^{\text{'}}}$ is the activity of Li ions at LiCoO_2_/SiO_2_ interface and ${a}_{{s}^{\text{'}}}$ is the activity of Li ions at SiO_2_/Si interface. According to Equation (16), ${c}_{{s}^{\text{'}}}=c(0,{t}_{1})$ and ${c}_{{s}^{\text{'}\text{'}}}=c({d}_{2},{t}_{1})$. The remaining reference values are $\bar{t}=0.4$, $T=298\,K$^30^. Assuming γ = 1, the diffusion potential (${V}_{d}$) is calculated to be −2.5 × 10^−3^ *V*, which is much less than *V*_*N*_, meaning that *V*_*d*_ can be neglected. Thus V_EMF_ ≈ V_N_, reference values are ${x}_{1}=0.7$, ${\rho }_{1}=2.5\,g/c{m}^{3}$, ${\rho }_{2}=2.648\,g/c{m}^{3}$, ${M}_{1}=97.87\,g/mol$, ${M}_{2}=60.086\,g/mol$, ${V}^{0}=1.4\,V$^13,21,31^. The simulated E_2_-d_2_ characteristics are presented in Fig. 6(c), ${E}_{2}$ decreases with increasing of the SiO_2_ thickness. Thus, SiO_2_ works as a Li ion trapping layer. When external voltage is removed, the SiO_2_ trapping layer decreases the total electromotive field of device and maintains the states resistances.

With external voltage, Li ions transport from LiCoO_2_ to SiO_2_ and Si layers, which can produce a V_EMF_ and exhibit a nanobattery-like behavior. The phenomenon is similar to the observation reported in other redox-based memristors^23,24,34,35^. It has also been demonstrated that V_EMF_ is dependent on the chemistry and the transport properties of the materials system^30,36,37^. Thus, Li ion trapping mechanism, especially the relationship between electromotive field and SiO_2_ thickness in different regions, may be also adapted to other devices based on LiCoO_2_ and SiO_2_ material system such as Li-ion batteries and Li-ion based transistors. The critical point is actually relative to thickness and Li ion concentration of LiCoO_2_ layer, may change with fabrication parameters of devices.

## Conclusion

The influence of SiO_2_ trapping layer on memristive behavior of Pt/LiCoO_2_/SiO_2_/Si stacks is investigated in terms of electrical properties, device stability and retention. For the LiCoO_2_ based memristors, the trapping layer benefits device retention. It is necessary to control the thickness of trapping layer to improve device properties. A model based on Nernst potential and diffusion potential is proposed to elucidate the Li ion trapping mechanism in SiO_2_ and two different relationships between the electromotive field and SiO_2_ thickness are found. According to this model, SiO_2_ trapping layer decreases the total electromotive field of device and hence prevents Li ions from migrating back to LiCoO_2_. These findings reveal the Li ion trapping mechanism of SiO_2_ in LiCoO_2_ based memristors and provide insights into the performance improvement of memristors and other devices based on LiCoO_2_ and SiO_2_.

## Supplementary information

supporting information

## Data Availability

The datasets generated and analysed during the current study are available from the corresponding author.
