# Supplementary material for: Lithium ion trapping mechanism of SiO2 in LiCoO2 based memristors
Source: Sci Rep. 2019 Mar 25;9:5081. doi: 10.1038/s41598-019-41508-3 (PMC6434038; doi:10.1038/s41598-019-41508-3)
Supplement: Supplementary file 1 — supporting information [file 41598_2019_41508_MOESM1_ESM.docx]

Supporting Information

Lithium ion trapping mechanism of SiO_2_ in LiCoO_2_ based memristors

Qi Hu^1^, Runmiao Li^1^, Xinjiang Zhang^1^, Qin Gao^1^,Mei Wang^1^, Hongliang Shi^1^, Zhisong Xiao^1^, Paul K. Chu^2^and Anping Huang^1^*

^1^ School of Physics, Beihang University, Beijing 100191, China

^2^ Department of Physics and Department of Materials Science and Engineering, City University of Hong Kong, Tat Chee Avenue, Kowloon, Hong Kong, China


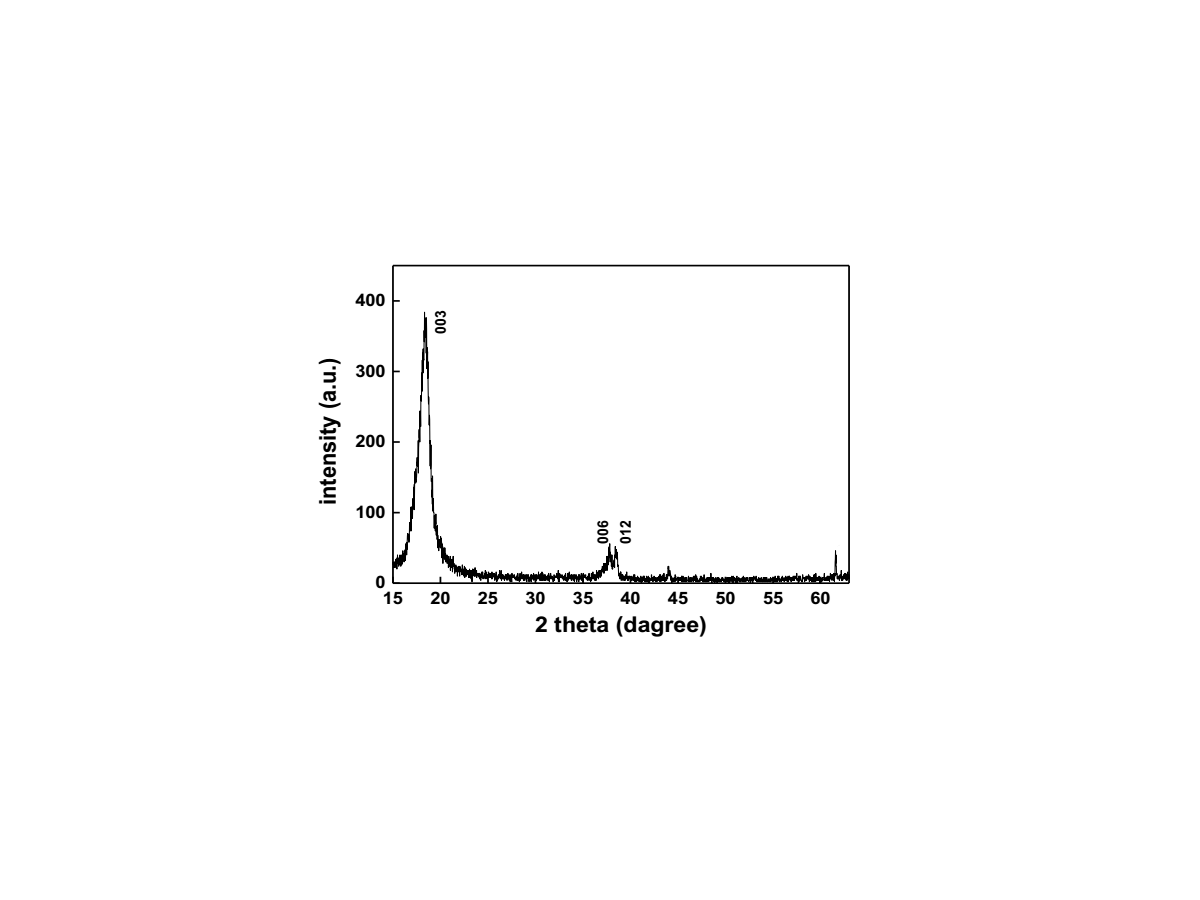


**Fig. S1.** XRD patterns of LiCoO_2_ fabricated at 550 ^o^C. The spectrum exhibits three peaks at 18°, 37.8° and 38.5° corresponding to the (003), (006) and (012) planes of the R-3m LiCoO_2_ phase, respectively.


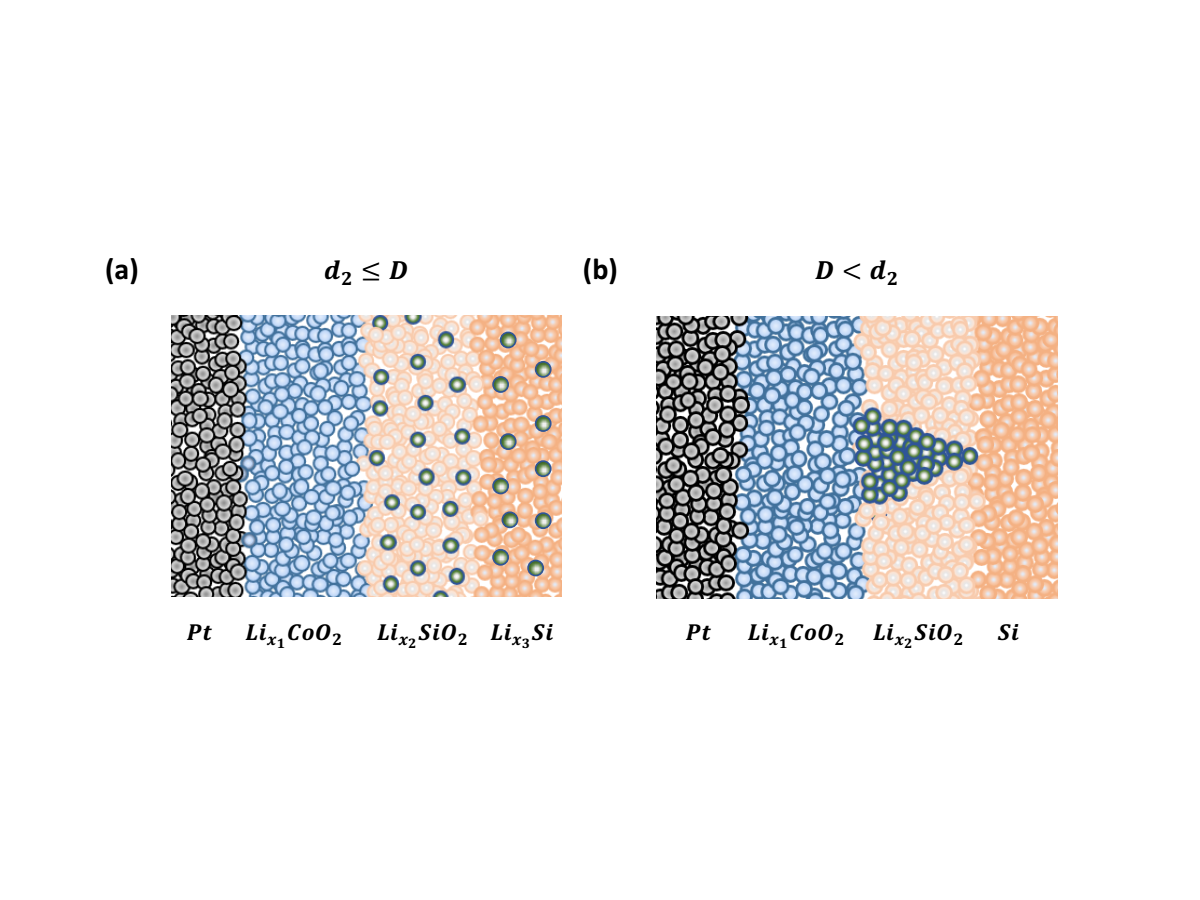


**Fig. S2.** Schematic illustration of the Li ions distribution in the Pt/LiCoO_2_/SiO_2_/Si stack: (a) SiO_2_ thickness ≤ D and (b) SiO_2_ thickness > D.

When d_2_ ≤ critical point (D), SiO_2_ undergoes fully lithiation and acts as a solid state electrolyte allowing Li ions to be transported to Si. The Li ions from LiCoO_2_ should be enough to ensure fully lithiation of SiO_2_. According to previous reports, the ranges of Li_x1_CoO_2_ and Li_x2_SiO_2_ are (0.6~0.9) and (1/3~2/3), respectively.^[1-3]^ Herein, x_2_ is the minimum (1/3) and x_1_ is the maximum (0.9) corresponding to supporting the minimum amount of Li ions. Thus,

$\frac{\rho_{1}d_{1}S\left( 1-x_{1}^{max} \right)}{M_{1}}\leq\frac{\rho_{2}DSx_{2}^{min}}{M_{2}}$ (S1)

When d_2_ > D，SiO_2_ is transformed to a resistive layer. Li ions mainly drift from LiCoO_2_ to SiO_2_ and negligible Li ions move to Si. Therefore, SiO_2_ undergoes partly lithiation. x_2_ is maximum (2/3) and x_1_ is minimum (0.6) corresponding to supporting the maximum amount of Li ions. Then,

$\frac{\rho_{1}d_{1}S\left( 1-x_{1}^{min} \right)}{M_{1}}\geq\frac{\rho_{2}DSx_{2}^{max}}{M_{2}}$ (S2)

The reference values are $\rho_{1}=2.5g/{cm}^{3}$, $\rho_{2}=2.648g/{cm}^{3}$, $M_{1}=97.87g/mol$, $M_{2}=60.086g/mol$. For $d_{1}=40nm$, the range of D is calculated to be 6.96 nm < D < 13.91 nm.


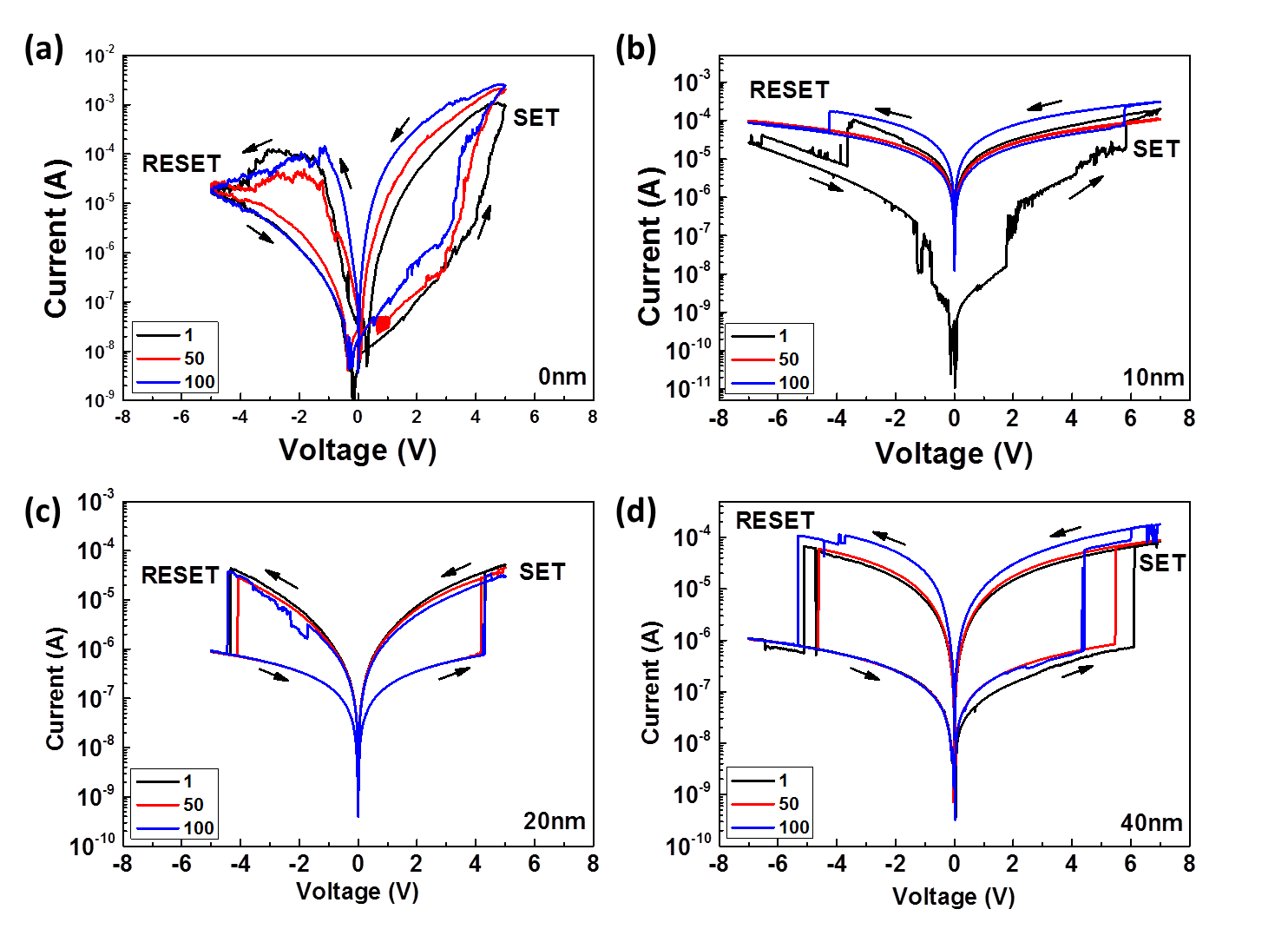


**Fig. S3.** I-V curves of samples with different SiO_2_ thicknesses during 128 cycles. (a) without SiO_2_ (b) 10 nm (c) 20 nm (d) 40 nm. Samples with 20 nm SiO_2_ exhibit the best repeatability.

**References**

[1] Milewska, A., Świerczek, K., Tobola, J. et al. The nature of the nonmetal–metal transition in Li_x_CoO_2_ oxide. Solid State Ionics. 263, 110-118 (2014).

[2] Marianetti, C. A., Kotliar, G., Ceder, G. A first-order Mott transition in Li_x_ CoO_2_. Nat. Mater. 3, 627 (2004).

[3] Ban, C., Kappes, B. B., Xu, Q. et al. Lithiation of silica through partial reduction. Appl. Phys. Lett. 100, 243905 (2012).
